# The Lyon-Cadett Method of Ventilation

**Published:** 1903-01-24

**Authors:** 


### THE LYON-CADETT METHOD OF VENTILATION.

The Camera Club, the members of which are chiefly interested in photography, arranges a series of lectures on scientific subjects during the winter session, and on Monday, the 12th inst., the subject was "Ventilation without Draughts." The lecturer was Mr. Thomas Glover Lyon, M.A., M.D. (Cantab), Physician to the City of London Hospital for Diseases of the Chest.

The large club room at 28 Charing Cross Road was occupied by about fifty members and friends, and the interesting feature of the evening was that the room was fitted up with the system of ventilation in question. This presented the appearance of a wooden frieze along one side of the room, in which were perpendicular slits a few inches apart, and two electric fans were fitted to the tops of the windows on the opposite side.

The system, as explained by Mr. Lyon by means of blackboard and lantern slides, is somewhat as follows.

### Description of the System.

The special feature is the employment of large inlets and outlets placed at opposite positions in a room. By this means "the air is admitted at a low velocity, and is conveyed to and from all parts of the room." The photographs showed what happens outside a room fitted with the Lyon-Cadett apparatus. There was first a filter, made by Mr. Lionel Teal. This consisted of a series of little oil baths, on the assumption that oil is a good medium for attracting dust. In the room in question, very little trouble was experienced in "dusting." Next was a heater, and an electric motor to drive the air into the conduit; the latter occupied the position of a frieze, inside the room, and running along one side. The space between the wall and the frieze may be guessed from the fact that a bookcase filled it where there would otherwise have been a recess. Perforated panels were placed below the frieze, through which the air was diffused into the room. In the club there were no panels, and the air had to pass through the perpendicular slits alluded to above. The panels exhibited were very decorative, and suggested a Moorish screen. At the back was a shutter which looked like the Venetian outside shutters so common in continental hotels; the slats being horizontal. From top to bottom were tapering "baffle-plates," or laths, which prevented the air from passing through the slats in too great a mass. The broad ends of the laths being at the bottom, the openings between the slats were thus made very narrow at the bottom of the panel, where the pressure of air would be greatest, but Mr. Lyon owned that too fine attention to this (a matter of conic sections) was unnecessary. So much for the inlet. The foul air is passed through extensive outlets in a similar manner into another conduit running along the opposite side of the room. The pamphlet put into the hands of the audience states that either the plenum or vacuum system of air propulsion may be used, or preferably a combination of both. "By employing blowers to force in the air and exhausts to draw it off, control of inlet and outlet is maintained without closing windows or doors, and open fireplaces can be used. The air on admission may be treated in any way desired."

The following example was given. The capacity of a room 50 feet long, 30 feet broad, and 20 high is 30,000 cubic feet, or 300 cubic feet per head for 100 persons. If we change the air in such a room once in six minutes, we supply 3,000 cubic feet to each person. Supposing we make the inlets and outlets in opposite side walls of the room equal in area to a quarter these walls, that is 250 feet in area, and pass air through the inlets and outlets at four inches a second, the air in the room will be changed every six minutes, and draughtless ventilation be effected with only 300 cubic feet of air space per head.

This was supplemented by two practical illustrations. Mr. Cadett stated that he had been present at a dinner-party in a room ventilated by this system, when about fifteen medical men were present, and that several of them tested their own pulse and respiration, and found both normal "after a good dinner and champagne." The room was 20 feet long, 15 feet wide, and 10 feet high, and the thermometer outside stood at 83 degrees. The second was more practical still. The club room, in which the majority of the men were smoking, was first reduced to its condition on "an ordinary evening" by shutting off the fan which supplied the conduit, and the two window fans, the windows being closed. The air was soon appreciably heavy and full of smoke; people began to cough, and in a few minutes there was a susceptible scent of lavender water which Mr. Lyon purposely introduced to show how smells lie about on a heavy atmosphere. A few minutes after the system was set going again, the room returned to pleasant atmospheric conditions.

### Theatres, Churches, and Assize Courts.

Several speakers remarked, in the discussion, that they had never spent an evening at the club with so little discomfort from foul and smoke-laden air. One (Dr. Williams) claiming to speak as the man in the street, said he was very fond of going to the theatre, but he very seldom went, because the ill-effects of the unventilated atmosphere in most of the London theatres required a day or two to recover from. He thought theatre managers ought to know about it, because he believed he represented a number of people, and it would be to the managers' great commercial advantage to ventilate their theatres properly. It meant many pounds in their pockets. If, as he understood, it cost about 1s. an hour to ventilate the club-room, it would not cost more than £1 or 30s. to make the average theatre tolerable.

He had sat in that club-room a great many times, but never in such comfort. Indeed the air was better than that of any room he had ever known.

Mr. Inwards, F.R.Met.S., thought the dining-room described by Mr. Lyon was, if anything, too well ventilated, in fact it would be sufficient for 200 people. As a guide to the proportion which the inlet should bear to the cubic content of the whole room, he suggested that of the orifice of the nostrils to the cubic content of the lungs; this, multiplied by the number of people, should, he thought, give the right result. For the rate at which the air should be admitted, he suggested that of human respiration.

The frequently stuffy condition of assize courts was urged by Mr. Herford, barrister; some such system, if applied especially to country courts, would, he said, be the greatest boon to his profession.

Churches also came in for their share of condemnation, and the well known "bank of fœtid air" that meets the person arriving fresh from out of doors was declared by the lecturer to be easily preventible by his method, even in country churches, where it could be applied by means of a gas-engine or an oil motor. "And the clergyman will pay," remarked the chairman, the Rev. C. E. Few, M.A.

The question of cost of course came up for discussion, and the lecturer remarked that it was wonderful how people got used to expense. At the outside, the apparatus in the club-room had cost £120; it was sufficient for 120 people, so that £1 per person might be taken as the first expense. Maintenance would be about 1s. an hour, including the heating apparatus kindly lent for this purpose by Mr. Dowsing.

### Hospitals.

The question of applicability to hospital wards was raised by Mr. E. W. Goodall, Medical Superintendent of the Eastern Fever Hospital, Homerton. He thought the methods of ward ventilation at present in use quite good enough, and did not think the expense of fans or other apparatus necessary. In the wards with which he was particularly concerned, the air was mechanically brought in from outside, and warmed by hot coils. There were fire-places in the middle of the wards, which acted as extractors; there were, as well, extractors in the walls. Provided that the wards were not overcrowded, a reasonable temperature was quite possible by these means. Although there were frequently very foul cases in the wards of a fever hospital, there was no offensive smell, even when the windows were closed, and in the depth of winter, with the full complement of cases, most of them in acute stages. He doubted whether it was necessary to go to "all this expense." Consider the ratepayer! If, however, Mr. Lyon could double the capacity of the wards, which were 120 by 24 feet, and 14 feet high, accommodating 20 patients each, so as to have 40 beds, the advantage would certainly be very great.

"Four times, easily," said Mr. Lyon at this point. In that case, Mr. Goodall replied, or even if they were only doubled, the initial expense would be nothing compared with the advantages to be gained.

The Chairman asked whether, if that were done, the number of patients would not greatly exceed that allowed by the Local Government Board? He had had a good deal to do with the building of hospitals, and he represented the requirements of the Local Government Board with regard to country districts, where large hospitals were demanded at great cost, and in places where the number of patients was small. How was it that during the Maidstone epidemic, when patients were much closer together than in ordinary times, the rate of mortality was only 7 or 8 per cent.? He did not advocate overcrowding, but he wanted small hospitals, which were easily kept in order, with a system whereby the number of patients could be doubled or even quadrupled, rather than large and expensive buildings. The cost saved to the rate-payer would be far in excess of the initial expense. Ventilation was far better than great empty rooms with still air in them.

In his reply, Mr. Lyon said a hospital ward should be treated in the same way as a crowded room. Patients were supposed to want more air than other people, and some almost idiotic regulations were made for them. To base the provision of air on the capacity of each person showed that people did not know what was wanted. The space required in many hospitals was entirely dependent on æsthetic principles—the patients liked to feel that they were not too near their neighbours. It was, for example, nice to have a tall room. Hospitals might be one fourth their present size, and yet be perfectly ventilated.

